# A Remote Raman System and Its Applications for Planetary Material Studies

**DOI:** 10.3390/s21216973

**Published:** 2021-10-21

**Authors:** Hongkun Qu, Zongcheng Ling, Xiaobin Qi, Yanqing Xin, Changqing Liu, Haijun Cao

**Affiliations:** 1 Shandong Key Laboratory of Optical Astronomy and Solar-Terrestrial Environment, School of Space Science and Physics, Institute of Space Sciences, Shandong University, Weihai 264209, China; quhongkun@mail.sdu.edu.cn (H.Q.); xiaobinqi@mail.sdu.edu.cn (X.Q.); yqxin@sdu.edu.cn (Y.X.); liucq@mail.sdu.edu.cn (C.L.); Caohj@mail.sdu.edu.cn (H.C.); 2 Key Laboratory of Space Active Opto-Electronics Technology, Chinese Academy of Sciences, Shanghai 200083, China

**Keywords:** remote Raman, planetary exploration, Raman spectra, anhydrous/hydrous minerals, organics

## Abstract

A remote Raman prototype with a function of excitation energy adjusting for the purpose of obtaining a Raman signal with good signal-to-noise ratio (SNR), saving power consumption, and possibly avoiding destroying a target by high energy pulses, which may have applications for Chinese planetary explorations, has been setup and demonstrated for detecting different minerals. The system consists of a spectrograph equipped with a thermoelectrically cooled charge-coupled device (CCD) detector, a telescope with 150 mm diameter and 1500 mm focus length, and a compact 1064 nm Nd:YAG Q-switched laser with an electrical adjusted pulse energy from 0 to 200 mJ/pulse. A KTP crystal was used for second harmonic generation in a 1064 nm laser to generate a 532 nm laser, which is the source of Raman scatting. Different laser pulse energies and integration time were used to obtain distinguishable remote Raman spectra of various samples. Results show that observed remote Raman spectra at a distance of 4 m enable us to identify silicates, carbonates, sulfates, perchlorates, water/water ice, and organics that have been found or may exist on extraterrestrial planets. Detailed Raman spectral assignments of the measured planetary materials and the feasible applications of remote Raman system for planetary explorations are discussed.

## 1. Introduction

As a powerful spectroscopic analysis technique, Raman spectroscopy that has been applied into many geoscientific areas including mineralogy, gemology, planetary analyses and space exploration, astrobiology and biomineralization, cultural heritage and archaeometry, etc., can provide accurate and detailed molecular and structural information of Earth and planetary materials. Owing to its advantages in no sample preparations, quick and non-destructive analyses, unambiguous phase identifications, as well as low-mass and robust behaviors on the mineralogy and mineral chemistries of rock and soil samples, many Raman spectroscopic studies on returned samples [1], meteorites [2] and planetary analogues [3] have been reported.

However, almost all of these studies used traditional micro-Raman with a working distance of several centimeters or less. During planetary exploration, tools with long detective distance are desirable. In the early 1960s, remote Raman was developed and employed in the detection of gases. Kobayasi and Inaba successfully observed Raman features of SO_2_ and CO_2_ from an oil smoke plume using a remote Raman system at a distance of 20 m [4]. In 1992, Angel employed a remote Raman system to identify solid inorganics and organics such as NaNO_3_, NaNO_2_, and acetaminophen at a distance of ten meters [5]. To date, several Raman systems have been proposed for either in situ or remote detection on planetary surfaces for lander or rover exploration missions to the Moon, Mars, Venus, Europa, asteroids, etc. Remote Raman systems for planetary exploration have been established by several groups [6,7,8,9], especially by the Raman spectroscopy laboratory at the University of Hawai’i, which has a greatly improved remote Raman instrument for the application in planetary explorations [8,10,11,12,13,14,15,16,17,18,19].

Wiens et al. detailed and introduced the SuperCam Instrument Suite including remote Raman spectrometer from the aspect of optical description, mechanical and thermal description, electronics, software, operation, and model development and environmental testing [20]. They provided valuable experience on developments of a remote Raman spectrometer in the planetary science field. More importantly, the green-laser (532 nm) remote Raman system with a detecting distance of up to 12 m was employed on the SuperCam on Mars2020/NASA, which successfully landed on Mars and was used to detect olivine and brighter Raman-emitting minerals for the first time in space [21,22]. ExoMars/European Space Agency (ESA) is planning to deploy a series of complementary analytical instruments (Pasteur Payload) including the Raman laser spectrometer (RLS) [23], which holds a 532 nm continuous laser source (20 mW). RLS is designed for identifying ultramafic igneous rocks and for establishing the presence of carbon (inorganic/organic) on Mars with a 15 mm working distance. All of these demonstrate that Raman spectroscopy is a proven and trusted technology that can provide highly accurate analytical results and can be applied to planetary explorations.

Laser power is an adjustable parameter in a traditional Micro-Raman spectrometer. Using proper laser power would obtain Raman signals with good SNR, but could also protect samples from being destroyed by high laser energy. Furthermore, many dark materials need higher laser energy than bright materials to excite Raman scattering. Hence, a remote Raman system with a function of excitation energy adjusting will not only obtain Raman signals of both brightness and dark materials without burning them but also reduce power consumption, which is significant while designing missions for planetary exploration. However, a remote Raman system with a function of excitation energy adjusting has been seldomly discussed.

Based on these laboratory studies and practical planetary exploration applications related to Raman spectrometers, herein, we aim to build a remote Raman system with a function of excitation energy adjusting. During planetary explorations, using different pulse energies for different types of minerals will help obtain Raman signal with good SNR, save power consumption, and possibly avoid destroying the target by high energy pulses. Raman peaks may be shifted by the environment of exoplanets; the calibration targets (e.g., minerals, organics, and diamond) carried with the rover from Earth are necessary for remote Raman to calibrate on extraterrestrial planets. The system we built showed an ability of obtaining hydrous/anhydrous minerals, water/water ice, and organics Raman spectra of high quality, which are comparable to the remote Raman spectra obtained by other groups. China has developed several successful missions to the Moon (Chang’e 1–5) and has an ongoing mission to Mars [24] (Tianwen 1), and will have further deep space exploration missions (e.g., Chang’e 6–8). Hopefully, our remote Raman system will be a desired candidate tool for new discoveries of further Chinese planetary explorations.

## 2. Experimental Setup and Samples

**Figure 1:**
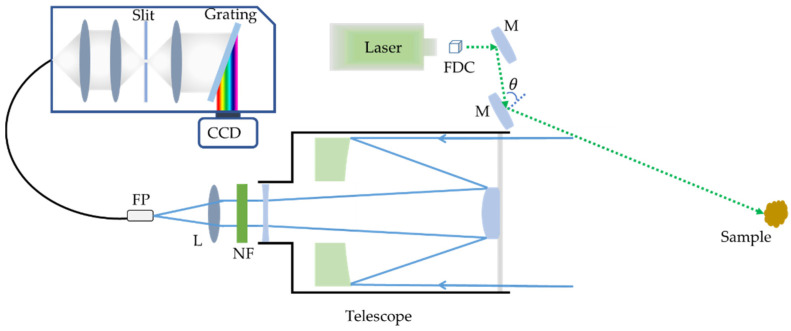
Schematic diagram of the remote Raman system. M, mirror; FDC, frequency doubling crystal; S, spectroscopy; FP, fiber probe; L, lens; NF = 532 nm Notch filter.

In this work, we built a remote Raman system in Shandong University using a compact Nd:YAG Q-switched laser source (Beamtech Optronics Co, Ltd. Company, Beijing, China. Dawa-200 Laser, 1064 nm, 0~200 mJ/pulse, 0~20 Hz, pulse width 9 ns, central laser spot divergence ~1 mrad, diameter ~6 mm), a spectrograph equipped with a CCD (Andor, DU416A-LDC-DD, Oxford Instruments Company, Oxford, UK) detector, and a telescope (CELESTRON NexStar 6SE, 150 mm diameter, 1500 mm focus length) shown in Figure 1. Commonly, shorter wavelength lasers will excite stronger Raman scattering because of the 1/λ^4^ increase in Raman scattering cross-sections; however, sample degradation or fluorescence may appear. Thus, 532 nm laser, which is generated by the second harmonic generation of 1064 nm laser using a frequency doubling crystal KTP (8 × 8 × 8 mm) with an exchange efficiency of 45–50%, was selected as the light source. The 532 nm green laser used in oblique geometry to excite Raman scattering was reflected onto samples by two 532 nm mirrors (45°). The oblique geometry delivers all the laser pulse power to the target and creates less near-field scattering at a short distance [10]. Laser spot diameter on samples, which is straight ahead of the telescope, is about 1.5 cm at a distance of 4 m. The laser energy on sample is about 0–65 mJ/pulse due to the energy loss caused by oblique geometry, which causes a larger angle of incidence (θ > 45°) shown in Figure 1.

When the laser hits the samples, Raman signal will be generated and collected by the telescope and then focused onto a fiber probe (FP) by the convex lens (L). The reflected 532 nm laser is removed by a 532 nm Notch filter (NF) fixed between the telescope and L. An optical fiber with 600 µm diameter core transferred the Raman signal into the spectrograph, in which two volume holographic gratings are employed. The spectral resolutions of the two volume holographic gratings with a 50 µm slit were 5.48 cm^−1^ in the wavelength region of 531–614 nm and 4.8 cm^−1^ in the wavelength region of 605–699 nm, respectively. Raman scattering was first focused to pass through a 50 µm slit by lens combination and then transmitted into a collimated beam by lens, finally irradiated on the volume holographic grating, which will disperse Raman scattering onto CCD. While conducting Raman scattering measurement, CCD worked in CW mode, which means the detector is “on” during the integration time. All of the Raman measurements were conducted in our lab at 4 m and 20 Hz with different pulse energies as well as different integration time in a dark environment for obtaining a better SNR.

With the purpose of testing the performance of this remote Raman system, we conducted a series of remote Raman measurements on minerals, analytical grade chemicals, deionized water, and water ice. Mineral samples (calcite, quartz, olivine, albite, K-feldspar) were collected by petrographic laboratory in Shandong University. No cutting or polishing was performed with these mineral samples. Analytical grade chemicals (e.g., epsomite (MgSO_4_·7H_2_O), KClO_4_, melanterite (FeSO_4_·7H_2_O), gypsum (CaSO_4_·2H_2_O), alunogen (Al_2_(SO_4_)_3_·18H_2_O), potassium nitrate (KNO_3_), potassium carbonate (K_2_CO_3_), NaClO_4_·H_2_O, Mg(ClO_4_)_2_·6H_2_O, L-Alanine, L-Phenylalanine, L-Glutamine and ethanol (C_2_H_6_O)) of analytical grade were purchased from the Sinopharm Chemical Reagent Beijing Co., Ltd. Rhomboclase (FeHSO_4_·4H_2_O) was synthesized using the method mentioned by Ling et al. [25]. We regarded these chemicals as pure samples. In order to obtain pure water ice, 70 mL deionized water held in a wide-mouth bottle of size 4.8 cm in diameter and 10.5 cm in height was placed into a freezer remaining at temperature around −20 °C for 2 h. No bubbles were found in water ice. All liquids and powder samples were measured through glass vials of size 2 cm in diameter and 5 cm in height with caps.

## 3. Results and Discussions

**Figure 2:**
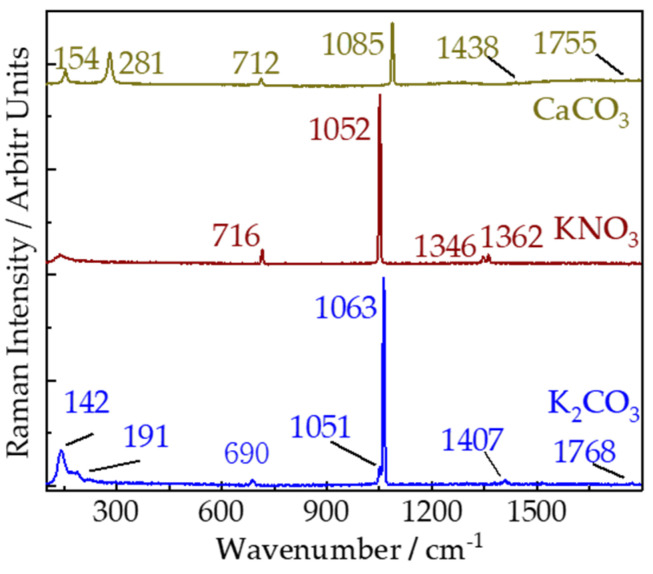
Remote Raman spectra of CaCO_3_, K_2_CO_3_, and KNO_3_ with 10 s integration time and 30 mJ/pulse laser (532 nm) incident to sample at 4 m target distance.

Figure 2 displays the remote Raman spectra of CaCO_3_, K_2_CO_3_, and KNO_3_ with 10 s integration time and 30 mJ/pulse laser (532 nm) incident to sample. According to previous works [26,27], Raman peaks of KNO_3_ in remote Raman spectrum can be identified as follows: the strongest Raman peaks 1052 cm^−1^ are assigned to ν_1_(A_g_) mode of NO_3_^−1^; another A_g_ mode observed at 1362 cm^−1^ is assigned to ν_3_ mode of NO_3_^−1^; peak at 1346 cm^−1^ belongs to ν_3_ (B_1g_) mode of NO_3_^−1^; peak at 716 cm^−1^ is attributed to ν_4_ (A_g_ + B_1g_) mode of NO_3_^−1^. Raman peaks in wavenumber region of 190–150 cm^−1^ and 310–285 cm^−1^ are attributed to relative translations between the cations and anionic groups [28]. Peaks at 690 and 712 cm^−1^ are asymmetric bending mode of ν_4_ of K_2_CO_3_ and CaCO_3_, respectively. Symmetric stretching mode of ν_1_ are observed at 1063 cm^−1^ for K_2_CO_3_ and at 1085 cm^−1^ for CaCO_3_. Asymmetric stretching mode of ν_1_ are identified by peaks near 1407 and 1755 cm^−1^ in remote Raman spectra of K_2_CO_3_ and CaCO_3_ respectively. An amount of 1786 cm^−1^ in K_2_CO_3_ and 1755 cm^−1^ in CaCO_3_ are ν_1_ + ν_4_ modes. Detailed assignments of Raman spectra of KNO_3_, CaCO_3_ and K_2_CO_3_ are shown in Table 1. Based on these remote Raman spectra, we can distinguish carbonates with different cations with ease. For the same cation (e.g., K), nitrates and carbonates share similarities in number of Raman peaks and their positions, although the stretching modes (ν_1_, ν_3_) of Raman peak seems to be lower for NO_3_ (1052 cm^−1^ and 1362 cm^−1^) than that of CO_3_ (1063 cm^−1^ and 1407 cm^−1^).

**Figure 3:**
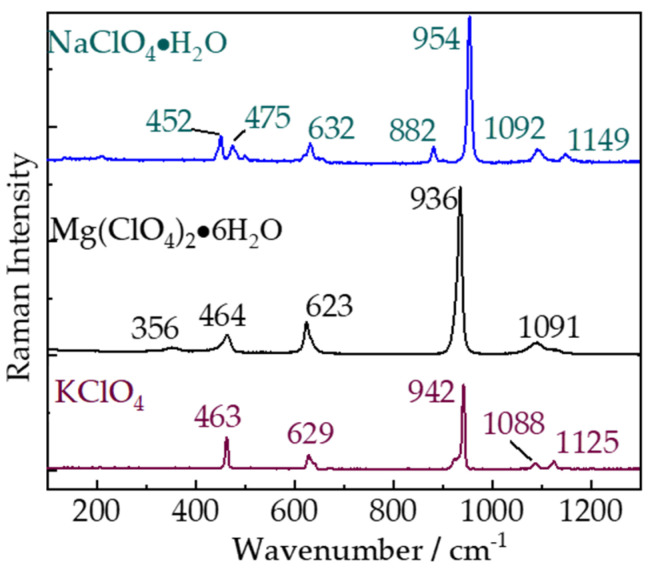
Remote Raman spectra of NaClO_4_⋅H_2_O, Mg(ClO_4_)_2_⋅ 6H_2_O, and KClO_4_ with 10 s integration time and 40 mJ/pulse laser (532 nm) incident to sample at 4 m target distance.

The remote Raman spectra of three perchlorates NaClO_4_·H_2_O, Mg(ClO_4_)_2_·6H_2_O, and KClO_4_ are shown in Figure 3. All major Raman peaks of these three perchlorates can be identified according to their remote Raman spectra obtained with 10 s integration time and 40 mJ/pulse laser (532 nm) incident to sample. Usually, perchlorates would show three main Raman bands, the symmetric Cl-O stretching mode (ν_1_ (A_1_)) in the wavenumber region of 950–930 cm^−1^, Raman active deformation (ν_4_ (T_2_)) of Cl-O between 635 and 625 cm^−1^, and a theoretical Raman inactive deformation (ν_2_ (E)) belonging to Cl-O within range of 470–445 cm^−1^. A theoretical inactive vibrational mode, which displays a negligible intensity as the fourth Raman band of perchlorates caused by the anti-symmetric stretching vibration (ν_3_ (T_2_)), was observed between 1150–1040 cm^−1^. Raman peak positions of KClO_4_, NaClO_4_·H_2_O, and Mg(ClO_4_)_2_⋅6H_2_O agree well with previous works [31] and are shown in Table 2 with vibrational modes. Perchlorates have been identified on Mars at the Phoenix landing site [32], Gale Crater [33], Viking sites [34,35], and in Martian meteorite EETA79001 [32] and ClO_4_^−1^ also has been found in Apollo lunar samples and carbonaceous chondrite meteorites [35]. The presence and distribution of perchlorate on Mars would have implications for Martian Cl cycles and the preservation of biosignatures [36]. Remote Raman with the ability of identifying perchlorates will be a desirable tool to reveal more perchlorates in our solar system.

**Figure 4:**
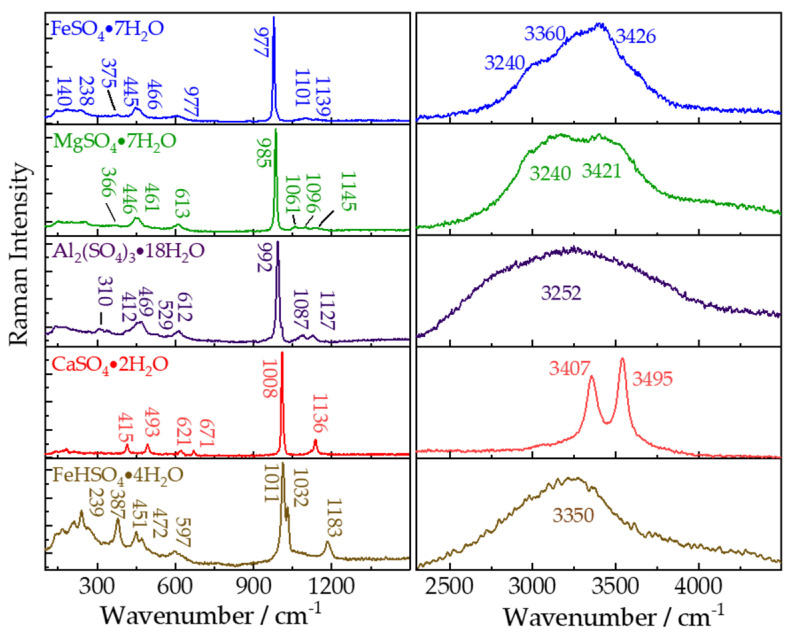
Remote Raman spectra of FeSO_4_⋅7H_2_O, MgSO_4_⋅ 7H_2_O, Al_2_(SO_4_)_3_⋅ 18H_2_O, CaSO_4_⋅ 2H_2_O, and FeHSO_4_⋅ 4H_2_O with 20 s integration time and 65 mJ/pulse laser (532 nm) incident to sample at 4 m target distance.

As a significant secondary mineral, sulfates may have important implications for past and present environmental evolutions of Mars. Different types of hydrous/anhydrous sulfates (e.g., Mg-, Ca-, Fe-, and Al-sulfates) have been found on Mars by orbital remote sensing and roving missions [37]. We conducted remote Raman measurements on five types of hydrous sulfates with 65 mJ/pulse laser (532 nm) incident to sample and 20 s integration time. As shown in Figure 4, the remote Raman system is able to clearly obtain the fingerprint Raman bands of FeSO_4_⋅7H_2_O, MgSO_4_⋅7H_2_O, Al_2_(SO_4_)_3_⋅18H_2_O, CaSO_4_⋅2H_2_O, and FeHSO_4_⋅4H_2_O from 100 to 4000 cm^−1^. The strongest peaks of five hydrous sulfates at 977, 985, 992, 1008, and 1101 cm^−1^ are caused by symmetrical stretching mode (ν_1_(SO_4_^2−^)) of SO_4_^2−^ ions in FeSO_4_⋅7H_2_O, MgSO_4_⋅7H_2_O, Al_2_(SO_4_)_3_⋅18H_2_O, CaSO_4_⋅2H_2_O, and FeHSO_4_⋅4H_2_O, respectively. Another ν_1_(SO_4_^2−^) mode of FeHSO_4_⋅4H_2_O was observed near 1032 cm^−1^. These frequency shifts of the strongest Raman peaks of covalent groups SO_4_ usually indicate different strong metallic ions (Fe, Mg, Ca, Al), which share the coordinating O with S, within their structure. As a result, the vibrational frequencies of S-O would be affected by different metallic ions because of their difference in atomic weights and attractive forces between the Fe, Mg, Ca, Al and O. The remote Raman spectra of these five hydrous sulfates caused by ν_3_(SO_4_^2−^) modes are different from each other. MgSO_4_⋅7H_2_O produces three peaks at 1061, 1096, and 1145 cm^−1^. FeSO_4_⋅7H_2_O has two peaks near 1101 and 1139 cm^−1^. CaSO_4_⋅2H_2_O and FeHSO_4_⋅4H_2_O display only one peak around 1136 and 1183 cm^−1^ respectively. Al_2_(SO_4_)_3_⋅18H_2_O has two peaks at 1087 and 1127 cm^−1^. The other two fundamental vibrational modes ν_2_ and ν_4_ of SO_4_^2−^ were found at: ν_2_-446 and 461 cm^−1^, ν_4_-613 cm^−1^ in MgSO_4_·7H_2_O; ν_2_-445 and 465 cm^−1^, ν_4_-618 cm^−1^ in FeSO_4_⋅7H_2_O; ν_2_-415 and 493 cm^−1^, ν_4_-621 and 671 cm^−1^ in CaSO_4_⋅2H_2_O; ν_2_-451 and 472 cm^−1^, ν_4_-597 cm^−1^ in FeHSO_4_⋅4H_2_O; ν_2_-412, 469 and 529 cm^−1^, ν_4_-612 cm^−1^ in Al_2_(SO_4_)_3_⋅18H_2_O. In shorter wavenumber region, the vibration of the (M, H_2_O) in MgSO_4_⋅7H_2_O is near 366 cm^−1^, in FeSO_4_⋅7H_2_O are at 140, 238, and 375 cm^−1^, in FeHSO_4_⋅4H_2_O are at 239 and 378 cm^−1^, and in Al_2_(SO_4_)_3_⋅18H_2_O is around 310 cm^−1^. Structural water in sulfates could produce different Raman bands. The O-H stretching modes have been observed at 3297 and 3421 cm^−1^ in MgSO_4_⋅7H_2_O, at 3240, 3360, and 3426 cm^−1^ in FeSO_4_⋅7H_2_O, at 3407 and 3495 cm^−1^in CaSO_4_⋅2H_2_O, at 3350 cm^−1^ in FeHSO_4_⋅4H_2_O, at 3252 cm^−1^ in Al_2_(SO_4_)_3_⋅18H_2_O. The detailed assignments have been concluded in Table 3. The ability of exact identification of hydrous salts would make it possible to acquire a more refined knowledge of past and/or present aqueous environment of extraterrestrial planets using remote Raman spectrometers.

**Figure 5:**
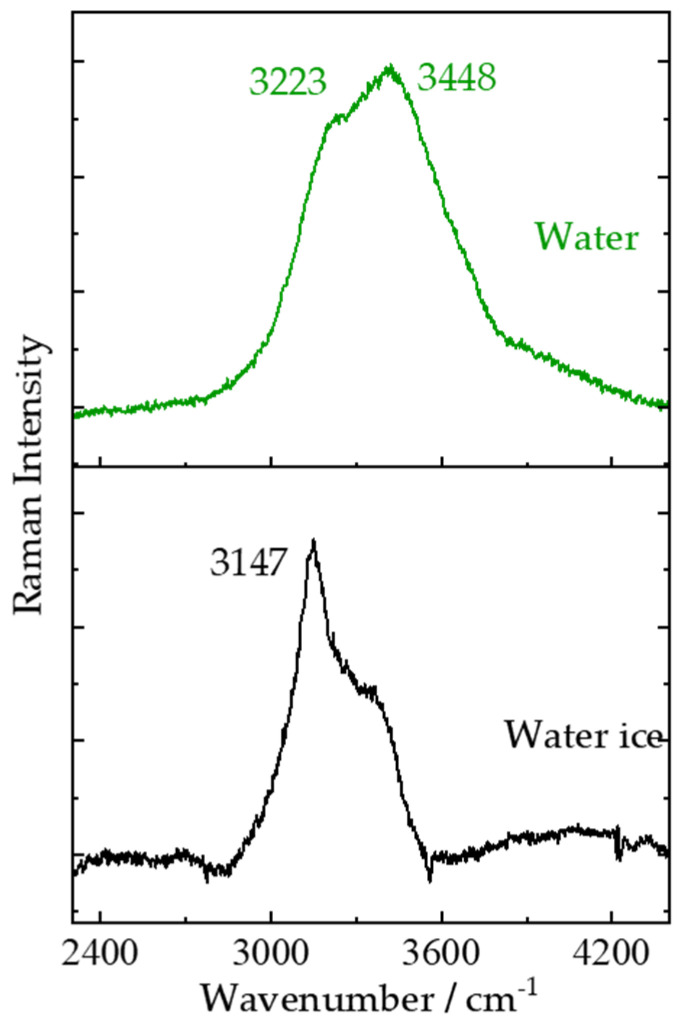
Remote Raman spectra of water and water ice with 20 s integration time and 65 mJ/pulse laser (532 nm) incident to sample at 4 m target distance.

Water displays strong Raman signals in longer wavenumber region due to intramolecular stretching such as the symmetric and antisymmetric stretching vibrational modes. In deionized water Raman spectra obtained at room temperature, two strong broad Raman bands were observed around 3223 and 3448 cm^−1^ which are attributed to the symmetric (ν_1_) and antisymmetric stretching (ν_3_) vibrational modes of the water molecule, respectively [40]. The increase of order in the ice structure will sharpen and shift the H-O-H symmetric stretching mode of water toward short wavenumber region. A sharper Raman band near 3147 cm^−1^ [41], which is clear enough to distinguish ice and water, was observed in remote Raman spectrum of water ice shown in Figure 5. Water is thought to be essential for supporting life; thus, the presence of water and/or water ice on a planet could make things easier for astronauts drinking and living, as well as in situ resource utilization for creation of oxygen and rocket hydrogen-oxygen fuel. Water ice on the surface of the Moon, Ceres, Mercury, Mars and other planets as well as moons has been reported mostly by visible and near infrared spectral remote sensing [42,43,44]. A remote Raman system carried on a rover would provide positive evidence for the presence and distributions of water ice, which is conductive to further exploration and utilization of water and/or water ice on these celestial bodies.

**Figure 6:**
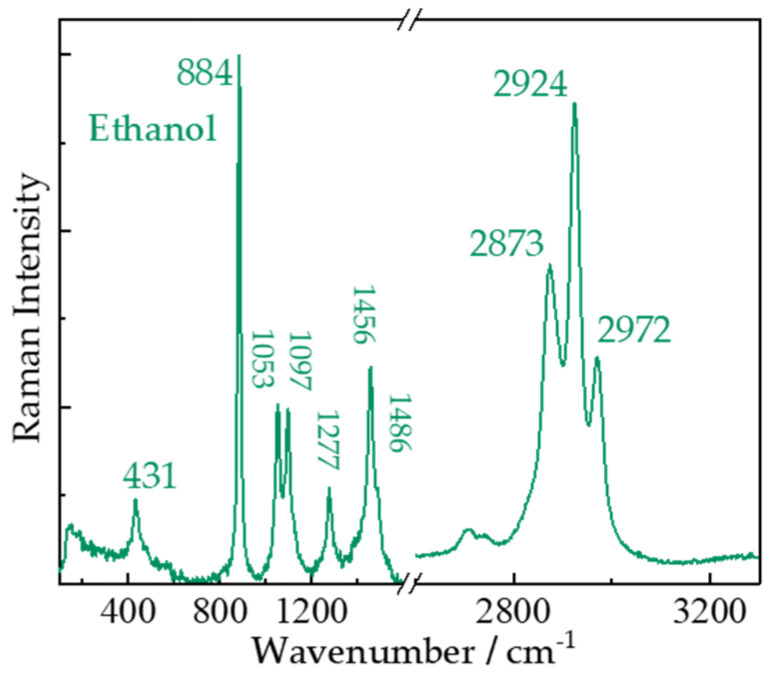
Remote Raman spectra of ethanol (C_2_H_6_O) with 15 s integration time and 50 mJ/pulse laser (532 nm) incident to sample at 4 m target distance.

**Figure 7:**
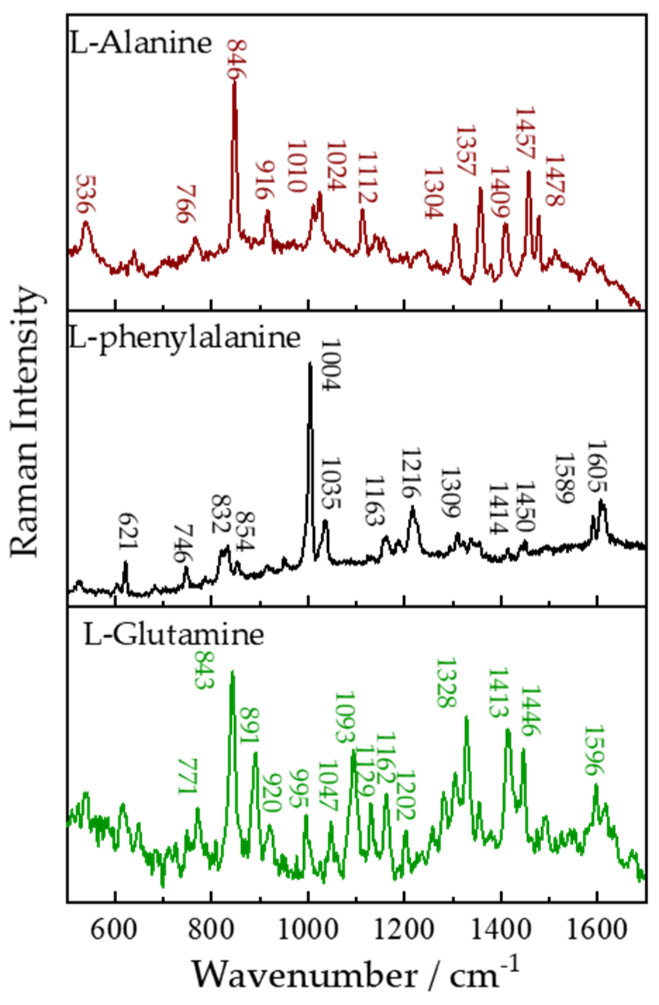
Remote Raman spectra of L-alanine, L-phenylalanine, and L-glutamine with 15 s integration time and 40 mJ/pulse laser (532 nm) incident to sample at 4 m target distance.

Organics, which might be indicative for life, are always attractive targets in planetary explorations. Definite detection of organics on extraterrestrial planets and moons might suggest the presence of life. We conducted remote Raman measurements on liquid ethanol (C_2_H_6_O), solid L-alanine (C_3_H_7_NO_2_), L-phenylalanine (C_9_H_11_NO_2_), and L-glutamine (C_5_H_10_N_2_O_3_), shown in Figure 6 and Figure 7. The weak peak at 431 cm^−1^ in Figure 6 is caused by bending vibration of CCO. The strongest peak at 884 cm^−1^ is attributed to stretching vibrational mode of CCO and peaks at 1053 and 1097 cm^−1^ are assigned to deformation mode of CCO. Peak around 1456 cm^−1^ is produced by bending vibrational modes of CH_3_ and CH_2_. The shoulder at 1486 cm^−1^ is bending vibrational mode of CH_3_.The symmetric stretching mode of CH_3_ is observed at 2873 cm^−1^. The symmetric and asymmetric CH_3_ stretching modes produce peaks at 2924 and 2972 cm^−1^, respectively [45]. We did not observe water peaks because of the 99% volume ratio of the ethanol.

L-alanine (C_3_H_7_NO_2_), L-phenylalanine (C_9_H_11_NO_2_), and L-glutamine (C_5_H_10_N_2_O_3_) serving as basic elements for the various proteins would display many Raman peaks due to many different kinds of vibrational modes. The Raman spectra of them are shown in Figure 7. For L-alanine, the strongest Raman peak 846 cm^−1^ is attributed to the (CCH_3_) symmetric stretching vibration, the 532 cm^−1^ peak is due to the rocking motion of the CO_2_^−^ group. The rocking motion of the CH_3_ group appeared at 1010 and 1024 cm^−1^. A Raman peak of 1112 cm^−1^ is attributed to CN stretching vibration and 1304 cm^−1^ is caused by CH deformation. The CH_3_ symmetric deformation band (1357 cm^−1^) and asymmetric deformation bands (1457 and 1478 cm^−1^) were also observed. In Raman spectra of L-Phenylalanine, the intense Raman peaks at 1004 cm^−1^ of L-Phenylalanine is caused by C-C symmetric stretching in ring. Peak at 621 cm^−1^ exhibits the presence of O-C=O in-plane bending modes. Wagging vibration of CH_2_ is observed near 832 cm^−1^. 854 cm^−1^ is assigned to NH_2_ deformation. 1035 cm^−1^ is distributed to C-H plane deformation in ring. Raman band corresponding to C-N stretching is observed at 1163 cm^−1^. Raman peaks at 1216, 1309, 1414, and 1450 cm^−1^ are, respectively, identified to ring breathing, CH_2_ wagging vibration, symmetric stretching vibration of COO−, and CH_2_ scissor vibration. C-C stretching in ring displays two Raman peaks around 1589 and 1605 cm^−1^. From Raman spectra of L-Glutamine, we can find that the ν(CN) vibrational band can be found at 843 cm^−1^ and the ν(CC) vibrational bands are at 995 and 1047 cm^−1^. The δ(CH_2_) rocking vibrations (891 cm^−1^) and δ(CH_2_) twist vibrations were also obtained. Raman peaks at 1093 and 1328 cm^−1^ are attribute to the symmetric ν(CN) stretching vibrational band and the CH deformation vibration. Two δ(NH_3_^+^) rocking vibrations were observed at 1129 and 1162 cm^−1^. The detailed assignments of these three organics are listed in [46]. The ability for distinguishing organics of our remote Raman system has been demonstrated by experiments with L-alanine, L-phenylalanine, and L-glutamine.

**Figure 8:**
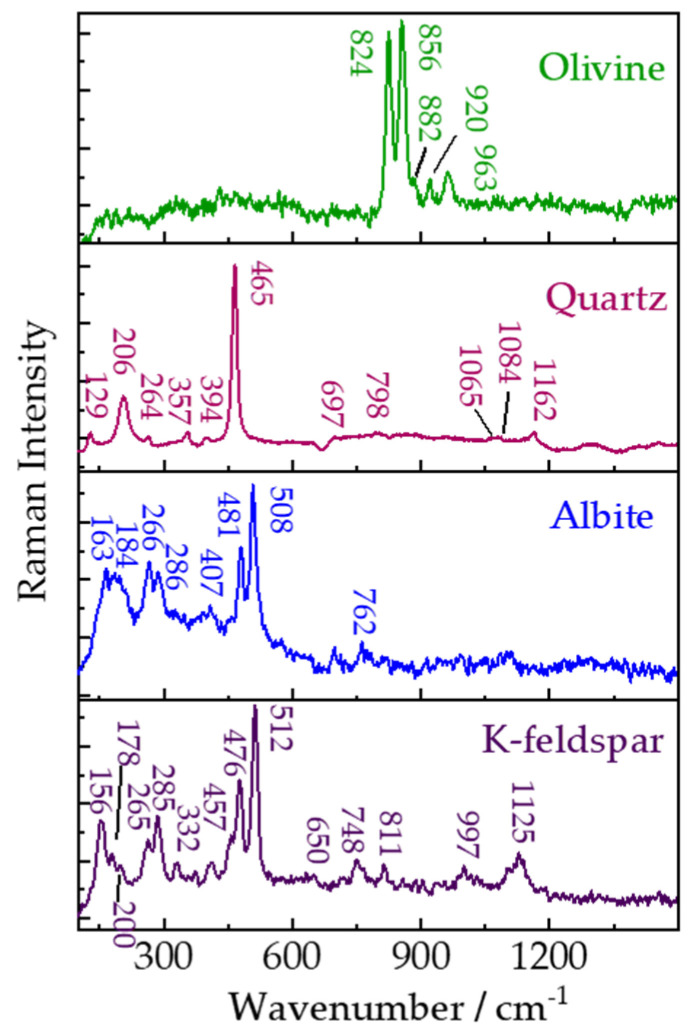
Remote Raman spectra of olivine, quartz, albite, and K-feldspar with 30 s integration time and 65 mJ/pulse laser (532 nm) incident to sample at 4 m target distance.

Figure 8 shows remote Raman spectra of olivine, quartz, albite, and K-feldspar with 30 s integration time and 65 mJ/pulse laser (532 nm) incident to sample. The dominant double Raman peaks occurring near 824 and 856 cm^−1^ are characteristic Raman features of olivine arising from coupled symmetric and asymmetric stretching vibrational modes of SiO_4_ tetrahedra. Raman spectra related to quartz have been investigated in detail under different pressures and temperatures in previous studies [47,48]. In our remote Raman spectra of quartz obtained at room temperature, Raman peaks from quartz agree well with that from α-quartz and are identified as the fundamental frequencies of A (206, 357, 465 and 1084 cm^−1^) and E (128, 264, 394, 697, 795–806, 1065 and 1162 cm^−1^) modes. Feldspars, which include different members, are the most abundant among all minerals and are thought to be a primary tool for classifying igneous rock. Raman spectra of feldspars should be similar with each other due to closely related members. Alkali feldspar series are between Na(Si_3_Al)O_8_ and K(Si_3_Al)O_8_, and the plagioclase feldspar series are between Na(Si_3_Al)O_8_ and Ca(Si_2_Al_2_)O_8_. It is a significant index whether remote Raman system is sufficiently sensitive to distinguish the small changes in structure and chemistry occurring between members of feldspars. K-feldspar and albite were selected as two feldspar members to conduct remote Raman measurement. In the region of 600 to 450 cm^−1^, the double Raman bands caused by a mixed Si-O-Si (or Si-O-Al) bending/stretching mode are observed at 481 and 508 cm^−1^ in albite, but triple characters near 457, 476 and 512 cm^−1^ are found in K-feldspar [49]. Comparing the Raman spectra of K-feldspar and albite collected by our system, we concluded that our remote Raman system is capable of identifying different feldspar members.

Raman features of olivine, albite, and K-feldspar are useable for determining mineralogy, providing mineral chemistries as well as aiding lithologic distinction [49,50]. The shift of the most intense Raman peak of quartz near 465 cm^−1^ has been used to estimate impact pressure [51], which would cause a distortion of the SiO_2_ structural framework. Applications for estimating impact pressure using this Raman peak shift have been demonstrated in planetary materials, e.g., lunar soils [52], lunar meteorites [53]. The instrument, which is individually capable of providing mineralogic, compositional information and impact pressure, is strongly and scientifically desired in planetary explorations.

**Figure 9:**
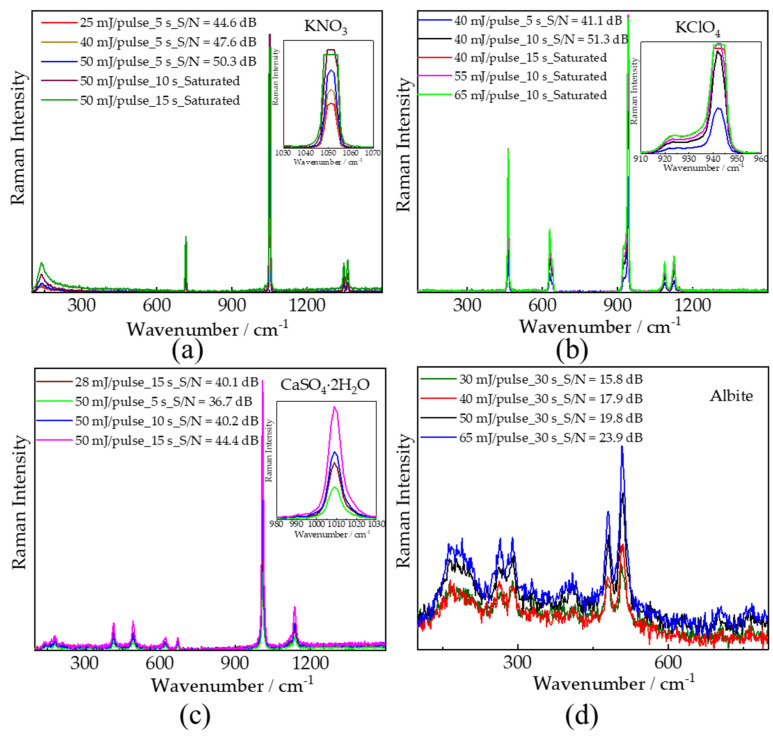
Remote Raman spectra of (**a**) KNO_3_, (**b**) KClO_4_, (**c**) CaSO_4_⋅2H_2_O and (**d**) Albite obtained with different pulse energies and integration time.

The Raman spectra of four samples (in Figure 9) were selected to demonstrate the experimental repeatability which is crucial for developing instrument. Figure 9 indicates that the repeatability of our remote Raman system is reliable. In Figure 9a, the SNR increases when increasing laser energy from 25 to 50 mJ/pulse with 5 s integration time and Raman signal got saturated with 10 and 15 s integration time as laser energy is 50 mJ/pulse. Similar results could be found in Figure 9b. We can also find that the SNR increases while prolonging integration time with same laser energy in Figure 9c and increases with the increase of laser energy with same integration time. In conclusion, stronger Raman peaks and higher SNR could be obtained by either prolonging integration time (Figure 9a–c) or increasing pulse energy (Figure 9d). However, some saturated Raman signal were observed in Figure 9a,b, e.g., 1052 cm^−1^ in KNO_3_ obtained with 50 mJ/pulse laser (532 nm) and 10–15 s integration time, 942 cm^−1^ in KClO_4_ obtained with 55–65 mJ/pulse laser (532 nm) and 10 s integration time. Thus, long integration time and/or high pulse energy would cause higher power consumption and unnecessary saturated Raman signal. Distinguishable Raman spectra can be obtained using 25 mJ/pulse laser (532 nm) and 5 s for KNO_3_, 40 mJ/pulse laser (532 nm) and 5 s for KClO_4_, 28 mJ/pulse laser (532 nm) and 15 s for CaSO_4_⋅2H_2_O, and 30 mJ/pulse laser (532 nm) and 30 s for albite in our experiment. It is not our goal to find out the minimum pulse energy/integration time for excitation of Raman signal for each sample, but to propose a remote Raman system with a function of excitation energy adjusting for reducing power consumption, obtaining Raman spectra with good SNR, and possibly avoiding damage of targets. The root mean square (RMS) SNR values shown in Figure 9 were calculated using formula below [54],

(1) $$\frac{S}{N}=\frac{I_{peak}-I_{background}}{N_{rms,\;background}}$$

The strongest peaks were selected as *I_peak_*, and *I_background_*, which were the strongest background intensity in a region where no Raman signal was detected. *N_rms, background_* is the RMS value of the region mentioned before. The RMS SNR values of some Raman peaks were not calculated due to the saturation that led to flat-top peaks with no accurate intensity value.

## 4. Conclusions

A remote Raman prototype working in oblique geometry was developed in Shandong University, aiming at providing suggestions on planetary exploration analysis techniques for Chinese planetary exploration. The Raman spectra acquired by our remote Raman system at a distance of 4 m demonstrates the ability of remote Raman for detecting silicates, carbonates, anhydrous/hydrous minerals, water/water ice, and organics. With its character of being sensitive to the molecular structure of a sample, Raman spectra enable us to identify the mineralogy and deduce the mineral chemical composition. For planetary mineralogy detection and survey, a remote Raman system has advantage on no sample preparations, quick and remote analyses, and unambiguous phase identifications that can help us identify minerals, organics, water/water ice, and other volatiles (e.g., CO_2_ and H_2_S) on the Moon, Mars, Venus, asteroids, and icy satellites, etc.

## Figures and Tables

**Table 1:** Assignments of Raman peaks of K_2_CO_3_, CaCO_3_, and KNO_3_ (in cm^−1^) [28,29,30].

K_2_CO_3_	CaCO_3_	KNO_3_	Assignments
142 191	154 281	137	T (K, CO_3_) T (Ca, CO_3_) B_1g_ (KNO_3_)
690	712	716	ν_4_-Asymmetric bending mode
1063	1085	1052	ν_1_-Symmetric stretching mode
1407	1438	1362 1364	ν_3_-Asymmetric stretching mode
1768	1755		ν_1_ + ν_4_

**Table 2:** Assignments of Raman peaks of KClO_4_, NaClO_4_⋅ H_2_O, and Mg(ClO_4_)_2_⋅ 6H_2_O (in cm^−1^) [31].

KClO_4_	${\mathrm{NaClO}}_{4}\cdot$H_2_O	$\mathrm{Mg}({\mathrm{ClO}}_{4}{)}_{2}\cdot$6H_2_O	Assignments
463	475 452	464	Deformation (ν_2_ (E))
629	632	623	Deformation (ν_4_ (T_2_))
942	954	936	Symmetric stretch (ν_1_ (A_1_))
1125 1088	1149 1092	1091	Anti-symmetric stretch (ν_3_ (T_2_))

**Table 3:** Assignments of Raman peaks of five hydrated sulfates (in cm^−1^) [25,38,39].

${\mathrm{MgSO}}_{4}\cdot$ 7H_2_O	${\mathrm{FeSO}}_{4}\cdot$ 7H_2_O	${\mathrm{CaSO}}_{4}\cdot$ 2H_2_O	${\mathrm{FeHSO}}_{4}\cdot$ 4H_2_O	${\mathrm{Al}}_{2}({\mathrm{SO}}_{4}{)}_{3}\cdot$ 18H_2_O	Assignments
366	140 238 375		239 378	310	T (Fe, H_2_O) T (Mg, H_2_O) T (Ca, H_2_O) T (Al, H_2_O)
446 461	445 466	415 493	451 472	412 469 529	ν_2_(SO_4_)
613	618	621 671	597	612	ν_4_(SO_4_)
985	977	1008	1011 1032	992	ν_1_(SO_4_)
1061 1096 1145	1101 1139	1136	1183	1087 1127	ν_3_(SO_4_)
3297 3421	3240 3360 3426	3407 3495	3350	3252	ν(H_2_O)

## Data Availability

Not applicable.
